# Contemporary Prevalence of Oral Clefts in the US: Geographic and Socioeconomic Considerations

**DOI:** 10.3390/jcm13092570

**Published:** 2024-04-27

**Authors:** Hilliard T. Brydges, Matteo Laspro, Alexandra N. Verzella, Andre Alcon, Jill Schechter, Michael F. Cassidy, Bachar F. Chaya, Eduardo Iturrate, Roberto L. Flores

**Affiliations:** 1 Hansjörg Wyss Department of Plastic Surgery, NYU Grossman School of Medicine, New York, NY 10017, USA; hilliard.brydges@nyulangone.org (H.T.B.); matteo.laspro@nyulangone.org (M.L.); alexandra.verzella@nyulangone.org (A.N.V.); andre.w.alcon@kp.org (A.A.); jwm2161@cumc.columbia.edu (J.S.); michaelfcassidy3@gmail.com (M.F.C.); bachar.chaya@vumc.org (B.F.C.); 2 Department of Medicine, NYU Grossman School of Medicine, New York, NY 10017, USA; eduardo.iturrate@nyulangone.org

**Keywords:** orofacial clefting, cleft lip, cleft palate, socio-economic status, geographic factors, incidence

## Abstract

**Background:** Socio-economic status, living environments, and race have been implicated in the development of different congenital abnormalities. As orofacial clefting is the most common anomaly affecting the face, an understanding of its prevalence in the United States and its relationship with different determinants of health is paramount. Therefore, the purpose of this study is to determine the modern prevalence of oral–facial clefting in the United States and its association with different social determinants of health. **Methods:** Utilizing Epic Cosmos, data from approximately 180 US institutions were queried. Patients born between November 2012 and November 2022 were included. Eight orofacial clefting (OC) cohorts were identified. The Social Vulnerability Index (SVI) was used to assess social determinants of health. **Results:** Of the 15,697,366 patients identified, 31,216 were diagnosed with OC, resulting in a prevalence of 19.9 (95% CI: 19.7–20.1) per 10,000 live births. OC prevalence was highest among Asian (27.5 CI: 26.2–28.8) and Native American (32.8 CI: 30.4–35.2) patients and lowest among Black patients (12.96 CI: 12.5–13.4). Male and Hispanic patients exhibited higher OC prevalence than female and non-Hispanic patients. No significant differences were found among metropolitan (20.23/10,000), micropolitan (20.18/10,000), and rural populations (20.02/10,000). SVI data demonstrated that OC prevalence was positively associated with the percentage of the population below the poverty line and negatively associated with the proportion of minority language speakers. **Conclusions:** This study examined the largest US cohort of OC patients to date to define contemporary US prevalence, reporting a marginally higher rate than previous estimates. Multiple social determinants of health were found to be associated with OC prevalence, underscoring the importance of holistic prenatal care. These data may inform clinicians about screening and counseling of expectant families based on socio-economic factors and direct future research as it identifies potential risk factors and provides prevalence data, both of which are useful in addressing common questions related to screening and counseling.

## 1. Introduction

Orofacial clefting (OC) is the most common congenital anomaly affecting the face [1,2]. OC, including isolated cleft lip (ICL), isolated cleft palate (ICP), and cleft lip with cleft palate (CLP), are highly variable in presentation. They require comprehensive multidisciplinary care from the neonatal age to facial maturity to fully restore form and function. Diagnosis is often made in obstetric and gynecologist check-ups and, if left untreated, OC can lead to significant impairments in aesthetics, hearing, oral health, and speech as well as psychosocial development [3]. Importantly, the consequences of OC on feeding can lead to undernutrition and even death [3,4]. Given the challenges and importance of early diagnosis, strong epidemiologic data may be useful in informing clinician screening practices.

The pathophysiology of non-syndromic OC is not well-understood and varies both globally and among different racial and ethnic groups [5]. Rates of craniofacial anomalies have been found to differ by geographic region with increased predominance in the Americas with rates in the United States remaining stable while there was a marked decline in other world regions [6]. OC has been associated with certain racial and ethnic groups, despite historic epidemiologic studies often examining populations with limited racial, ethnic, and socio-economic diversity [5,7,8].

The Center for Disease Control (CDC) estimates the prevalence of CLP, ICL, and ICP as 1 in 1600 (6.25 in 10,000), 1 in 2800 (3.57 in 10,000), and 1 in 1700 (5.88 in 10,000), respectively [9]. However, these estimates likely fail to capture the highly variable OC prevalence across a diverse US population. Additionally, due to large ethnic, racial, and socio-economic status (SES) diversity in the US, international studies conducted in relatively homogenous populations may not be representative of the US population.

To implement interventions aimed at promoting equitable care, a thorough understanding of the US prevalence of OC as well as geographic and sociodemographic factors associated with OC is crucial. Therefore, this study aims to better define the US prevalence, identify the geographic variability, and clarify the impact of sociodemographic factors on OC.

## 2. Methods

To identify previous epidemiologic data related to OC, a narrative review of PubMed was conducted by two independent reviewers (ML and HB). Studies that analyzed primary OC data and reported prevalence rates were gathered. Data related to OC prevalence were extracted along with measures of statistical confidence and study-specific considerations and limitations.

The primary data analyzed in this study were sourced from Cosmos^TM^ (EPIC Systems, Verona, WI, USA), a data collective that amalgamates and de-identifies data from over 180 participating institutions throughout the USA that utilize EPIC medical records. Cumulatively, these data account for over 169 million patients, spanning all 50 states, including rural and urban populations. Owing to their large sample size, these data closely reflect the demographics of the USA when compared to US Census data (Supplemental Figure S1). Before analysis, the data undergo multiple levels of quality and fidelity assessments both within contributing institutions and the Cosmos platform [10]. This multi-step process involves standardization of values for categorical data, verification of calculated data, removal of identifiable and redundant data, and combination and deduplication of medical records across institutions.

Patients born between 3 November 2012 and 2 November 2022 were included in this study. At the time of analysis, aggregated data were sourced directly from the Cosmos pre-built interface (SlicerDicer^TM^, EPIC Systems, Verona, WI, USA), in which categorical variables are reported as counts and continuous variables are reported as means and standard deviations.

In this study, eight cohorts of OC patients were identified using a combination of International Classification of Disease (ICD) codes. These eight cohorts are not mutually exclusive as patients could have multiple craniofacial anomalies. A complete list of ICD codes used for cohort identification can be found in Table 1. Following cohort identification, descriptive analyses of demographic variables including race, sex, ethnicity, regional and temporal prevalence trends, and social determinant associations were conducted. 

The Social Vulnerability Index (SVI) was used to identify social determinants of health among the included cohorts (Supplemental Figure S2). SVI is a zip code-based measure, defined by the CDC to aid with the identification of at-risk communities and thereby improve equitable allocation of resources in the event of disasters [11]. Owing to its utility, SVI is now commonly used as a geographic proxy for social determinants of health [12]. SVI is derived using a combination of variables, which are broadly categorized into four CDC-defined themes: minority/language, household composition, socioeconomic status, and housing/transportation. Cumulative SVI (accounting for all variables) and the four major themes (each accounting for a unique subset of variables) are then normalized. The result is a zip code-associated percentile value, where higher percentiles indicate more vulnerable communities. In this study, cumulative SVI, as well as the four themes, and the individual composition variables were analyzed independently. Analysis was conducted both by OC cohort and, to control for potential confounding, by racial and ethnic subgroups. 

Prevalence rates per 10,000 live births were calculated along with 95% confidence intervals. In addition, univariate analysis was employed to assess the associations between the abovementioned variables and the eight OC cohorts. Student *t*-tests assessed the differences in annual prevalence rates between the first and final years of analysis. Cochrane–Armitage tests were used to evaluate for differences in trends of ordinal SVI variables. All statistics were conducted using R (Version 4.1.3) and a *p*-value less than or equal to 0.05 was considered significant.

## 3. Results

Following a scoping review of the literature, a previously published meta-analysis of 69 primary studies on orofacial clefting prevalence was examined. In this study, the pooled prevalence of ICL was 0.30 per 1000 live births, ICP was 0.33 per 1000 live births, and CLP was 0.45 per 1000 live births [5].

**Figure 1:**
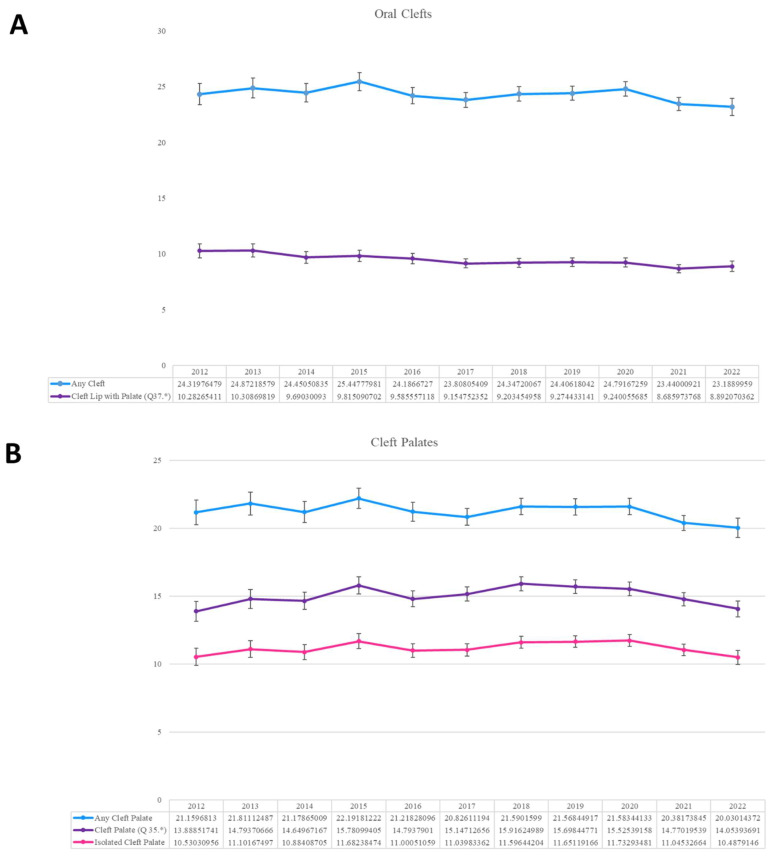

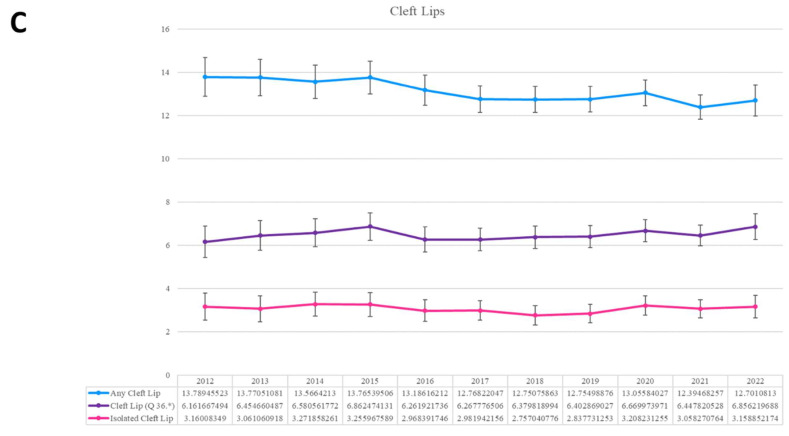
Annual trends in oral cleft prevalence over 10 years of study. (**A**) Overall oral clefts, (**B**) cleft palates, and (**C**) cleft lips. * = statistically significant difference.

Regarding the Epic Cosmos portion of the study, there were 15,697,366 patients identified between November 2012 and November 2022, of which 31,216 patients were diagnosed with OC, for a prevalence rate of 19.9 (95% confidence interval (CI): 19.7–20.1) per 10,000 live births (Table 1). Overall, any cleft palates (ACP) were at least twice as common as any cleft lips (ACL) (18.2 CI: 18.0–18.4 vs. 9.6 CI: 9.4–9.7), while ICP was nearly five times more common than ICL (10.3 CI: 10.1–10.5 vs. 2.0 CI: 1.9–2.1). Annual trends in prevalence rates demonstrated no significant difference over time in all cohorts, save for CLP which exhibited a statistically significant decrease in prevalence comparing 2012 with 2022 (10.3 CI: 9.7–10.9 vs. 8.9 CI: 8.4–9.4, *p* < 0.001) (Figure 1).

**Figure 2:**
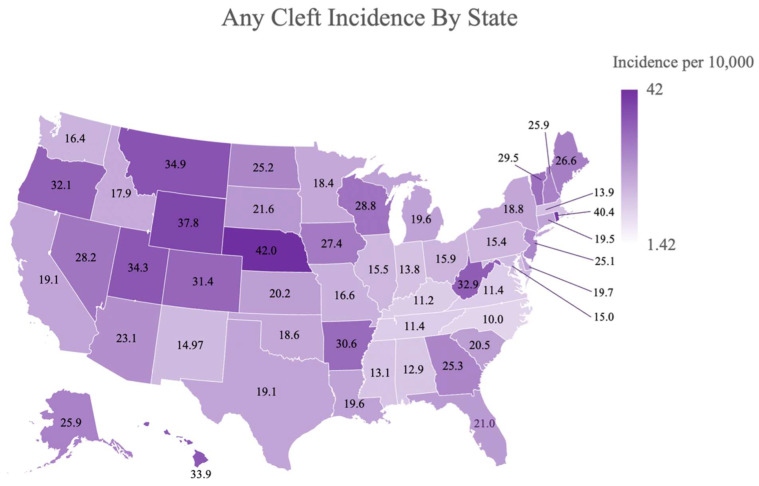
US Map with the prevalence of all oral clefts by states.

When examining geographic associations, the prevalence of OC varied considerably between states. Nebraska exhibited the highest rate of OC at 42.0 per 10,000 live births, closely followed by Rhode Island and Wyoming at 40.4 and 37.0, respectively. The states with the lowest prevalence of OC were Virginia, Kentucky, and North Carolina with 11.4, 11.2, and 10.0 oral clefts per 10,000 live births, respectively. Figure 2 provides a visual comparison of state-level prevalence.

Other studies have reported that CL varies among ethnic populations but CP appears to be stable across ethnic populations [7,13,14]. We found that the prevalence of CP varied across races and ethnic populations, while CL is more homogenous between groups. Additionally, we found that the overall prevalence of OC was observed to be highest among Asian (27.5 CI: 26.2–28.8) and Native American (including native Hawaiian, Alaskan, and Pacific Islanders) patients (32.8 CI: 30.4–35.2) and lowest among Black patients (12.96 per CI: 12.5–13.4). Males and Hispanic patients exhibited higher OC prevalence than female and non-Hispanic patients (Table 2). There were no differences in prevalence rates among metropolitan (20.23 per 10,000), micropolitan (20.18 per 10,000), and rural (20.02 per 10,000) populations (Table 2).

SVI analysis examining all racial and ethnic groups combined found the prevalence of OC correlated with household composition (theme 1) and type (theme 3) (Table 3). Household compositions, comprising a greater number of disabled and pediatric individuals, all correlated with increased OC prevalence. Greater OC prevalence also correlated with a higher proportion of mobile homes and crowding both within homes and communities (population density). Communities with a higher proportion of minority language speakers are associated with decreased OC prevalence.

In recognition that race and ethnicity are social constructs and that socioeconomic findings are modulated by historical inequities related to these groups, SVI analysis was stratified into three racial/ethnic groups (White, Black, and Hispanic). Results were largely similar to the unstratified cohort (Table 4). Notably, however, socioeconomic metrics, including uninsured status, poverty, and unemployment, were correlated with OC primarily among White and Hispanic patients while these variables were less significant among Black patients. Furthermore, the negative correlation between minority language speaking and OC was most notable among Hispanic-identified patients.

## 4. Discussion

The national prevalence for CLP, ICL, and ICP was calculated using validated medical records from the Epic Cosmos database, which captured over 15 million live births between 2012 and 2022. This represents approximately 38% of the newly born population of the United States during that period. Our data found a higher prevalence of ICP (1 in every 971 babies) and CLP (1 in every 1398 babies) than the previously published data from Mai et al. (2019) and that which was cited by the CDC (1 in every 1700 and 1 in every 1600 babies, respectively) [9]. However, we found a lower prevalence of ICL (1 in every 4978 babies) than the previous estimates from Mai and colleagues (1 in every 2800 babies) [9]. The variable prevalence in cleft palate is particularly remarkable considering the historical divergence from the previously reported data in which cleft palate remained stable across different populations. In one meta-analysis, heterogeneity in reporting cleft palate prevalence was I^2^ = 99.9%, indicating very high data divergence in methods among the 59 studies included studies [5]. As OC has been found to correlate with geography and local environments, result divergence is expected. Yet, given the divergence within American-produced data, methodological differences between studies must cofound reported prevalence. As the data presented here emerged from a compilation of geographically and socioeconomically diverse spaces in the United States, this study provides a more accurate assessment of prevalence. This is because previously published data over-emphasized large metropolitan areas or specific geographic regions [9,15]. When not stratifying by race and ethnicity, the SVI theme that correlated most positively with OC is household composition. In the aggregate cohort, increasing socio-economic status or SVI did not correlate with increased OC; however, when the data were stratified by race and ethnicity, a positive correlation was observed.

In this study, no difference was found in the prevalence of OC between metropolitan (>50 k), micropolitan (10–50 k), and rural/small town (<10 k) towns (Table 2). This contradicts a previous study conducted in the state of Washington between 1989 and 2014, which found that infants born to mothers in rural settings had an increased odds (OR of 1.12, including SE or standard deviation) of having a cleft compared to infants born to mothers in urban settings even after adjusting for race and ethnicity [16]. This is further emphasized by the difference in prevalence between the three most agriculturally productive states. While the third highest producer, Nebraska, had the highest prevalence found (42.0), California and Iowa, the highest and second highest producers, respectively, appear to have mid-range prevalence compared to the averaged national prevalence (19.1 and 27.4, respectively). Similarly, environmental exposures associated with more urban areas, such as ambient air pollutants, have also been found to positively correlate with OC development [17,18].

Although this study’s findings contrast with previous publications, the national comparison of rural and urban regions may not provide enough data detail to investigate regional toxic exposures. This is especially true provided that the types and quantities of toxins likely vary within each rural and urban setting. For instance, different pesticides are required for different crops. In turn, urban pollutants may vary based on the prevalent industries in each metropolitan area. As such, while the study did not find differences between the two environments, that is not to say that teratogens produced from urban air pollutants or fertilizers might not individually negatively impact in utero cleft development. Therefore, more targeted epidemiologic studies followed by translational studies are warranted to assess the effects of individual toxins on cleft development. Some possibilities for these OC differences include variations in specialty and community hospital proportions represented in the Epic Cosmos across states and variations in specific environmental exposures and at-risk populations across states. However, the reasons for this considerable geographic discrepancy are beyond the scope of this analysis and remain an important area of necessary future investigation.

Social determinants of health have long been linked with an increase in all-cause congenital abnormalities [19]. Factors include access to medical care, maternal nutritional level, and increased exposure to hazardous living conditions. SVI is a robust and broadly utilized method of assessing social determinants of health and has been previously employed to identify social risk factors for other congenital anomalies [20]. In this study, SVI correlation with the undifferentiated OC cohort yielded mixed results. However, stratifying patients by race and ethnicity revealed that SVI correlated with increased OC development. In terms of examining individual variables, the percent below the poverty line had the strongest correlation with OC. Given the complex implications of poverty, the factors affecting this potential relationship warrant further exploration; however, in the interim, this finding may help inform future screening initiatives.

While strides have been made towards health equity through the Medicaid program and the Affordable Care Act, our analysis and previous studies have found that insurance status is predictive of certain OC pathologies [21,22]. While there has been advocacy to increase maternal insurance during pregnancy, a considerable number of women undergo insurance churn or lose insurance postpartum [23,24]. In fact, maternal insurance status has been found to negatively correlate with post-natal health visits [22,25]. Given the multidisciplinary nature and complexity of OC care, parents’ ability to navigate the healthcare system and insurance coverage may play an integral role in OC outcomes.

Interestingly, our data indicate that communities with a higher proportion of non-English language speakers exhibited less association with OC. To the knowledge of the investigators, this metric has not been previously studied. While a non-English language could be seen as a barrier to healthcare access and navigation, speaking a minority language promotes community creation and has been linked to improvements in living status especially among minoritized communities [26,27]. In fact, cultural outpouching has been demonstrated to improve health outcomes. For instance, Makuau et al. (2016) noted the importance of the development of culturally pertinent community-centered health initiatives in Hawaii in improving health outcomes in the Native Hawaiian population [28]. Promoting similar policies in other minorized communities, including Native Americans and Hispanic populations, may promote similar outcomes concerning the mitigation of OC risks [29]. In 1999, the CDC started the Racial and Ethnic Approaches to Community Health, which were intended to address the health problems most directly affecting communities by bolstering community partnerships [29,30]. Therefore, the incorporation of OC education through community health initiatives in populations that are found to have an increased prevalence of OC could help to address this disparity.

Finally, examination of the effects of household composition and housing/transportation yielded mixed findings, which were less consistent when patients were sub-grouped by race. While notable positive associations include disabled household members, living in mobile homes and crowding may portend potential environmental exposures. Overall, greater research is needed to elucidate these nuanced relationships.

## 5. Strengths and Limitations

To the authors’ knowledge, this is the largest cohort study assessing the prevalence of OC in the United States as well as the most comprehensive analysis of the social determinants of health on OC development. However, the study does not come without its limitations. Firstly, while the EPIC Cosmos represents several public and private US hospitals, patients of other health systems not utilizing EPIC were inevitably excluded from the study. Similarly, while assessments of social determinants were captured, causations cannot be drawn from the study at hand. Hence, further prospective investigation of each factor is encouraged. Furthermore, additional limitations stem from the granularity of data available for analyses. To protect patient privacy, data analyzed in this study were only available when reported in aggregate. Thus, patient-level data could not be individually assessed, precluding multivariate analyses, which would have better clarified the relative significance of each social determinant. Moreover, SVI data are associated with a patient’s home zip code. Therefore, they may not directly reflect the social determinants of each patient. Future analyses should validate the findings of this study using individual-level data, such as patient-reported questionnaires. Lastly, although certain socio-determinants are pervasive in nations of different socio-economic standings, others must be analyzed from local populations to ensure public policies are community-oriented and locally focused.

## 6. Conclusions

In conclusion, this study examines the largest cohort of oral cleft patients reported to date and reports the contemporary US OC prevalence rates, which demonstrate an increase in prevalence from previous estimates. The prevalence of cleft palates appears to vary across ethnic populations, in contrast to previous reports. Importantly, we found that the percentage below the poverty line is strongly associated with OC, reinforcing the consequences of social determinants of health. While the geographic associations here investigated may be pertinent to an American population alone, the impact of SES on orofacial clefting can be applied to an international population. Thus, the findings presented here can help national and foreign providers to counsel expectant families, direct future research, and promote holistic prenatal care that focuses on parental socio-economic needs.

## Figures and Tables

**Table 1:** Incidence of Oral Clefts (per 10,000 births). Contains prevalence rates for the eight oral cleft cohorts analyzed in this study. ICDs used to identify each cohort were included along with cohort sample counts and 95% confidence intervals for calculated prevalence.

	ICD-10 Codes		Count	Incidence	95% CI
All Patients	Included	Excluded	15,697,366	-	-
All Cleft Lips	Q35. *, Q36. *, Q37. *		31,216	19.89	(19.67–20.11)
Any Cleft Palate	Q35. *, Q37. *		28,602	18.22	(18.01–18.43)
Cleft Palate (ICD)	Q35. *		22,297	14.20	(14.02–14.39)
Isolated Cleft Palate	Q35. *	Q36. *, Q37. *	16,154	10.29	(10.13–10.45)
Any Cleft Lip	Q36. *, Q37. *		15,062	9.60	(9.44–9.75)
Cleft lip (ICD)	Q36. *		8286	5.28	(5.16–5.39)
Isolated Cleft Lip	Q36. *	Q35. *, Q37. *	3154	2.01	(1.94–2.08)
Cleft palate with cleft lip (Q37. *)	Q37. *		11,232	7.16	(7.02–7.29)

* 0.1–0.9.

**Table 2:** Oral Cleft Incidences per Demographic Group. Prevalence of oral cleft cohorts, per 10,000 live births. Data are subdivided by race, ethnicity, sex assigned at birth, and population density.

	Any Cleft	Any Cleft Palate	Cleft Palate (Q 35. *)	Isolated Cleft Palate	Any Cleft Lip	Cleft Lip (Q 36. *)	Isolated Cleft Lip	Cleft Lip with Palate (Q37. *)
	Incidence (95% CI)	Incidence (95% CI)	Incidence (95% CI)	Incidence (95% CI)	Incidence (95% CI)	Incidence (95% CI)	Incidence (95% CI)	Incidence (95% CI)
Race								
White	24.44 (24.11–24.77)	22.01 (21.69–22.32)	17.87 (17.59–18.15)	13.02 (12.77–13.26)	11.42 (11.20–11.65)	6.44 (6.27–6.60)	2.43 (2.33–2.53)	8.41 (8.21–8.60)
Black	12.96 (12.53–13.40)	11.78 (11.37–12.19)	9.55 (9.18–9.92)	6.76 (6.45–7.08)	6.20 (5.90–6.50)	3.64 (3.41–3.87)	1.18 (1.05–1.31)	4.65 (4.39–4.91)
Asian	27.48 (26.16–28.79)	25.24 (23.98–26.50)	19.10 (18.01–20.20)	11.85 (10.99–12.72)	15.62 (14.63–16.61)	7.38 (6.70–8.06)	2.24 (1.86–2.61)	12.72 (11.83–13.61)
Native	32.82 (30.43–35.20)	30.20 (27.92–32.49)	24.66 (22.59–26.72)	17.18 (15.45–18.90)	15.64 (14.00–17.29)	8.43 (7.22–9.64)	2.61 (1.94–3.29)	12.22 (10.76–13.67)
Ethnicity								
Hispanic	21.73 (21.17–22.29)	19.90 (19.37–20.44)	15.40 (14.93–15.88)	10.45 (10.06–10.84)	11.28 (10.87–11.68)	6.31 (6.01–6.61)	1.82 (1.66–1.99)	8.91 (8.56–9.27)
Sex								
Male	21.28 (20.96–21.59)	18.89 (18.59–19.19)	14.50 (14.24–14.76)	10.07 (9.86–10.29)	11.20 (10.97–11.43)	6.23 (6.06–6.40)	2.38 (2.28–2.49)	8.27 (8.08–8.47)
Female	18.38 (18.07–18.69)	16.92 (16.62–17.21)	13.89 (13.62–14.16)	10.54 (10.30–10.77)	7.84 (7.64–8.04)	4.24 (4.10–4.39)	1.60 (1.51–1.69)	5.83 (5.66–6.00)
Location								
Metropolitan	20.23 (19.95–20.52)	18.17 (17.90–18.44)	14.39 (14.15–14.63)	10.44 (10.24–10.65)	9.79 (9.59–9.99)	5.38 (5.23–5.53)	2.06 (1.97–2.15)	7.23 (7.06–7.41)
Micropolitan	20.18 (19.57–20.78)	18.13 (17.55–18.70)	14.51 (13.99–15.02)	10.51 (10.07–10.95)	9.67 (9.25–10.09)	5.36 (5.05–5.67)	2.05 (1.86–2.24)	7.13 (6.77–7.49)
Rural/Small Town	20.02 (19.57–20.46)	18.04 (17.61–18.46)	14.42 (14.05–14.80)	10.39 (10.07–10.72)	9.62 (9.31–9.93)	5.32 (5.09–5.54)	1.98 (1.84–2.12)	7.16 (6.90–7.43)

Note: Metropolitan, population is more than <50,000 people, Micropolitan is 10,000–50,000, and Rural/Small town is <10,0000 people. * 0.1–0.9.

**Table 3:** Social Vulnerability Index Data (All populations). General SVI data analysis, including all oral cleft patients. Red + indicates that the diseased group tended to exhibit a statistically significant greater association with increasing social determinant burden. Green - indicates that the non-diseased group tended to exhibit a statistically significant greater association with increasing social determinant burden. N.S. signifies no statistical significance.

	Any Cleft	Any Cleft Palate	ICD Cleft Palate (Q 35. *)	Isolated Cleft Palate	Any Cleft Lip	ICD Cleft Lip (Q 36. *)	Isolated Cleft Lip	ICD Cleft Lip with Palate (Q37. *)
Social Vulnerability Index (SVI) (Overall)	-	-	-	+	+	+	-	+
Household Composition (Theme)	+	+	+	n.s.	+	+	n.s.	+
% > 65 years-old	+	+	+	+	+	+	+	+
% < 17 years-old	+	+	n.s.	-	+	+	n.s.	+
% Disabled	+	+	+	+	+	+	+	+
% Single Parent Home	-	-	-	-	-	-	-	-
Housing/Transport (Theme)	-	-	-	-	n.s.	n.s.	-	-
% Multi-Unit	-	-	-		-	n.s.	n.s.	-
% Mobile Homes	+	+	n.s.	+	+	+	+	+
% Crowding	+	n.s.	-	-	+	+	-	+
% No Vehicles	-	-	-	-	-	-	-	-
% Group Quarters	n.s.	n.s.	n.s.	n.s.	n.s.	+	n.s.	n.s.
Minority/Language (Theme)	-	-	-	-	-	-	-	-
% Minority	+	+	-	-	-	-	-	-
% Limited English Speaking	-	-	-	-	+	n.s.	-	n.s.
Socioeconomic (Theme)	-	-	-		+	+	n.s.	+
% Below Poverty	-	-	-	-	n.s.	n.s.	-	+
% Unemployed	-	-	-	-	n.s.	n.s.	-	-
% Without Highschool Diploma	-	-	-	-	+	+	-	+
% Uninsured	n.s.	n.s.	-	-	-	+	n.s.	+

*p* < 0.05; n.s. = no significant correlation; * 0.1–0.9.

**Table 4:** Social Vulnerability Index Data Stratified by Racial and Ethnic Groups. Subgroup analyses of SVI data. Cohorts were analyzed separately to control for race. Red (+) indicates that the diseased group tended to exhibit a statistically significant greater association with increasing social determinant burden. Green (-) indicates that the non-diseased group tended to exhibit a statistically significant greater association with increasing social determinant burden.

White Race	Any Cleft	Any Cleft Palate	ICD Cleft Palate (Q 35.*)	Isolated Cleft Palate	Any Cleft Lip	ICD Cleft Lip (Q 36. *)	Isolated Cleft Lip	ICD Cleft Lip with Palate (Q37. *)
Social Vulnerability Index (SVI) (Overall)	+	+	+	n.s.	+	+	n.s.	+
Household Composition (Theme)	+	+	+	n.s.	+	+	n.s.	+
% > 65 years-old	+	+	n.s.	n.s.	+	n.s.	+	n.s.
% < 17 years-old	n.s.	+	n.s.	n.s.	+	+	n.s.	+
% Disabled	+	+	+	+	+	+	+	+
% Single Parent Home	n.s.	n.s.	-	-	+	n.s.	-	+
Housing/Transport (Theme)	+	+	+	n.s.	+	+	n.s.	+
% Multi-Unit	-	-	-	-	-	-	-	-
% Mobile Homes	+	+	+	+	+	+	+	+
% Crowding	+	+	n.s.	-	+	+	n.s.	+
% No Vehicles	-	n.s.	-	-	n.s.	n.s.	n.s.	n.s.
% Group Quarters	+	+	+	+	+	n.s.	n.s.	+
Minority/Language (Theme)	-	-	-	-	n.s.	n.s.	-	n.s.
% Minority	-	-	-	-	n.s.	+	-	+
% Limited English Speaking	-	-	-	-	n.s.	n.s.	-	-
Socioeconomic (Theme)	+	+	+	n.s.	+	+	n.s.	+
% Below Poverty	+	+	n.s.	n.s.	+	+	n.s.	+
% Unemployed	+	+	n.s.	n.s.	+	+	n.s.	+
% Without Highschool Diploma	+	+	n.s.	-	+	+	n.s.	+
% Uninsured	n.s.	+	n.s.	-	+	+	n.s.	+
Black Race								
Social Vulnerability Index (SVI) (Overall)	n.s.	+	+	n.s.	n.s.	n.s.	n.s.	n.s.
Household Composition (Theme)	+	+	+	n.s.	+	n.s.	n.s.	+
% > 65 years-old	+	+	+	+	n.s.	n.s.	n.s.	n.s.
% < 17 years-old	n.s.	n.s.	n.s.	n.s.	+	+	n.s.	+
% Disabled	n.s.	n.s.	n.s.	n.s.	n.s.	n.s.	n.s.	n.s.
% Single Parent Home	n.s.	n.s.	n.s.	n.s.	n.s.	n.s.	-	n.s.
Housing/Transport (Theme)	n.s.	n.s.	+	+	-	n.s.	n.s.	n.s.
% Multi-Unit	-	-	-	n.s.	n.s.	n.s.	n.s.	n.s.
% Mobile Homes	+	+	+	+	+	+	n.s.	+
% Crowding	n.s.	n.s.	n.s.	n.s.	n.s.	n.s.	n.s.	n.s.
% No Vehicles	n.s.	n.s.	n.s.	n.s.	-	-	n.s.	n.s.
% Group Quarters	n.s.	n.s.	n.s.	+	n.s.	-	n.s.	n.s.
Minority/Language (Theme)	n.s.	n.s.	n.s.	n.s.	n.s.	n.s.	-	n.s.
% Minority	n.s.	n.s.	n.s.	-	n.s.	n.s.	n.s.	n.s.
% Limited English Speaking	n.s.	n.s.	n.s.	n.s.	n.s.	n.s.	n.s.	n.s.
Socioeconomic (Theme)	n.s.	+	+	n.s.	n.s.	n.s.	n.s.	n.s.
% Below Poverty	n.s.	n.s.	n.s.	n.s.	n.s.	n.s.	n.s.	n.s.
% Unemployed	n.s.	n.s.	n.s.	n.s.	n.s.	n.s.	n.s.	n.s.
% Without Highschool Diploma	n.s.	n.s.	n.s.	n.s.	n.s.	n.s.	n.s.	n.s.
% Uninsured	+	+	+	n.s.	+	+	n.s.	+
Hispanic Ethnicity								
Social Vulnerability Index (SVI) (Overall)	n.s.	n.s.	n.s.	-	+	+	n.s.	+
Household Composition (Theme)	n.s.	n.s.	n.s.	n.s.	+	+	n.s.	+
% > 65 years-old	n.s.	n.s.	n.s.	n.s.	n.s.	n.s.	n.s.	n.s.
% < 17 years-old	+	+	+	n.s.	+	+	n.s.	+
% Disabled	n.s.	n.s.	n.s.	n.s.	n.s.	n.s.	+	n.s.
% Single Parent Home	-	-	-	-	n.s.	n.s.	n.s.	n.s.
Housing/Transport (Theme)	n.s.	n.s.	n.s.	-	+	n.s.	n.s.	+
% Multi-Unit	-	-	-	-	-	-	n.s.	-
% Mobile Homes	+	+	+	+	+	+	n.s.	+
% Crowding	n.s.	n.s.	n.s.	-	+	+	n.s.	+
% No Vehicles	-	-	-	-	-	-	n.s.	-
% Group Quarters	n.s.	n.s.	n.s.	n.s.	n.s.	n.s.	n.s.	n.s.
Minority/Language (Theme)	-	-	-	-	n.s.	n.s.	-	n.s.
% Minority	-	-	-	-	n.s.	n.s.	n.s.	+
% Limited English Speaking	-	-	-	-	n.s.	n.s.	n.s.	n.s.
Socioeconomic (Theme)	+	+	n.s.	n.s.	+	+	n.s.	+
% Below Poverty	n.s.	n.s.	n.s.	-	+	+	n.s.	+
% Unemployed	n.s.	n.s.	n.s.	-	+	+	n.s.	+
% Without Highschool Diploma	n.s.	n.s.	n.s.	-	+	+	n.s.	+
% Uninsured	n.s.	n.s.	n.s.	n.s.	+	n.s.	n.s.	+

*p* < 0.05; n.s. = no significant correlation. * 0.1–0.9.

## Data Availability

Data can be shared upon request and following approval by the senior author.

## Supplementary Materials

The following supporting information can be downloaded at https://www.mdpi.com/article/10.3390/jcm13092570/s1. Supplemental Figure S1. Comparison of demographic, insurance, and social vulnerability between Cosmos patients and the US Census. Adapted from https://Cosmos.epic.com/ (accessed on 1 August 2023). Supplemental Figure S2. SVI sub-groups. Supplemental Table S1. ICD-10-CM codes are used to identify Oral Cleft Cohorts. Supplemental Table S2. Four main SVI categories are divided by subgroup.
