# Supplementary material for: Contemporary Prevalence of Oral Clefts in the US: Geographic and Socioeconomic Considerations
Source: J Clin Med. 2024 Apr 27;13(9):2570. doi: 10.3390/jcm13092570 (PMC11084882; doi:10.3390/jcm13092570)
Supplement: Supplementary file 1 [file jcm-13-02570-s001.zip › jcm-2971483-supplementary.pdf]

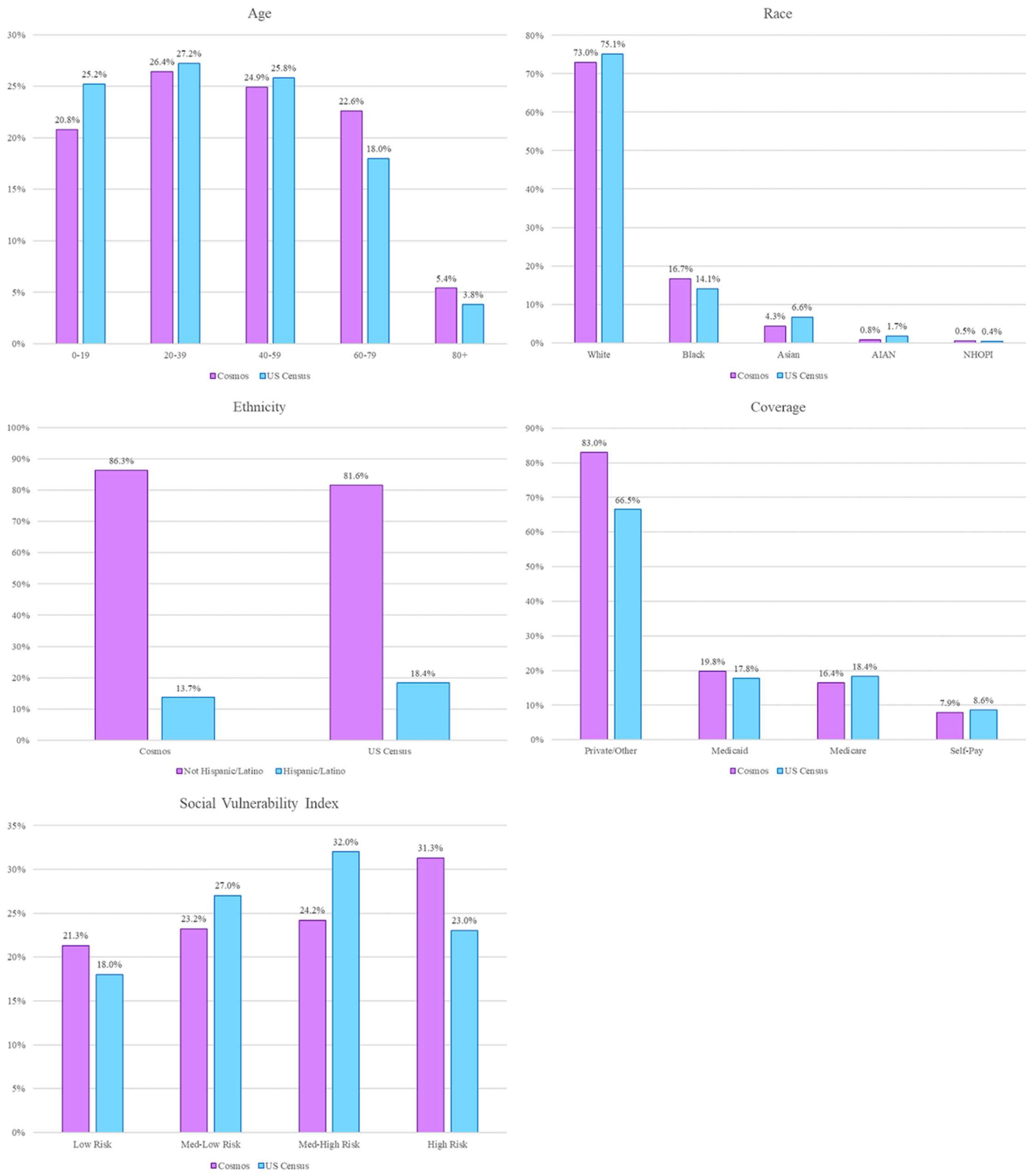

Supplemental Figure S1. Comparison of demographic, insurance, and social vulnerability between COSMOS patients and the US Census. Adapted from https://Cosmos.epic.com/.

**Socioeconomic status**	Below poverty<br>Unemployed<br>Income<br>No high school diploma
**Household composition & disability**	Aged 65 or older<br>Aged 17 or younger<br>Older than age 5 with a disability<br>Single-parent households
**Minority status & language**	Minority<br>Speaks English “less than well”
**Housing type & transportation**	Multi-unit structures<br>Mobile homes<br>Crowding<br>No vehicle<br>Group quarters

Supplemental Figure S2. SVI sub-groups.

Supplemental Table S1. ICD-10-CM codes are used to identify Oral Cleft Cohorts.

	ICD-10-CM	
**Cohort**	**Included**	**Excluded**
Any Oral Cleft (AOC)	Q35.* + Q36.* + Q37.*	-
Any Cleft Palate (ACP)	Q35.*+ Q37.*	-
Isolated Cleft Palate (ICP)	Q35.*	Q36.* + Q37.*
Any Cleft Lip (ACL)	Q36.* + Q37.*	-
Isolated Cleft Lip (ICL)	Q36.*	Q35.*+ Q37.*
Cleft Lip and Palate (CLP)	Q37.*	-

Supplemental Table S2. 4 main SVI categories divided by subgroup

**Socioeconomic status**	Below poverty<br>Unemployed<br>Income<br>No high school diploma
**Household composition & disability**	Aged 65 or older<br>Aged 17 or younger<br>Older than age 5 with a disability<br>Single-parent households
**Minority status & language**	Minority<br>Speaks English "less than well"
**Housing type & transportation**	Multi-unit structures<br>Mobile homes<br>Crowding<br>No vehicle<br>Group quarters
